# RAGE-Mediated Inflammation, Type 2 Diabetes, and Diabetic Vascular Complication

**DOI:** 10.3389/fendo.2013.00105

**Published:** 2013-08-21

**Authors:** Yasuhiko Yamamoto, Hiroshi Yamamoto

**Affiliations:** ^1^Department of Biochemistry and Molecular Vascular Biology, Kanazawa University Graduate School of Medical Sciences, Kanazawa, Japan

**Keywords:** rage, obesity, inflammation, toll-like receptors, pattern-recognition receptors

## Abstract

Obesity is associated with inflammation and type 2 diabetes. Innate immune system comprised of cellular and molecular components plays an important role in the inflammatory reactions. Immune cells like macrophages and their cell surface pattern-recognition receptors (PRRs) are representative for innate immunity promoting inflammatory reactions. The receptor for advanced glycation end-products (RAGE) is a member of PRRs and a proinflammatory molecular device that mediates danger signals to the body. The expression of RAGE is observed in adipocytes as well as immune cells, endothelial cells, and pancreatic β cells under certain conditions. It has been reported that RAGE is implicated in adipocyte hypertrophy and insulin resistance. RAGE-mediated regulation of adiposity and inflammation may attribute to type 2 diabetes and diabetic vascular complications.

Obesity is associated with an increased risk of developing type 2 diabetes, fatty liver disease, hypertension, and vascular complications (1). Proinflammatory and anti-inflammatory bioactive molecules produced from adipose tissues, known as adipokines, contribute to the burden of obesity-related diseases (2). Adipose tissue consists of heterogeneous populations of adipocytes, stromal preadipocytes, immune cells, and vascular cells, and it can respond rapidly and dynamically to alterations in nutrient excess caused by enhanced food consumption through adipocyte hypertrophy and hyperplasia (3). This results in a local inflammation in adipose tissue that propagates an overall systemic but chronic low-grade inflammation associated with the development of obesity-related comorbidities such as type 2 diabetes and cardiovascular diseases (2).

## Innate Immunity and RAGE-Mediated Inflammatory Reactions

The innate immune system can act as a double-edged sword in protecting the host against foreign enemies and destroying tissues via inflammation. It may represent an evolutionary strategy adopted by multicellular organisms to prevent the survival of cells that would otherwise cause more disastrous consequences in the individuals and their descendants. Toll-like receptors (TLR) and receptor for advanced glycation end-products (RAGE) can participate in innate immunity maintaining a delicate balance between clearance of pathogens and induction of exaggerated inflammatory responses.

Receptor for advanced glycation end-products is originally identified for recognizing advanced glycation end-products (AGE) (4). RAGE belongs to the immunoglobulin superfamily, and is now known as a member of pattern-recognition receptors (PRRs) and as a proinflammatory device. RAGE recognizes a variety of endogenous and exogenous ligands, including AGE, advanced oxidation protein products, high-mobility group box protein 1 (HMGB1), calcium-binding S100 proteins, β2-integrin Mac-1/CD11b, amyloid β peptide/fibril, lipopolysaccharide (LPS), phosphatidylserine, C1q, and lysophosphatidic acid (LPA) (5). It has been hypothesized that RAGE engagement of such ligands causes diabetic vascular complications, atherosclerosis, cancer, neurodegeneration, and inflammatory diseases (6). Anti-RAGE antibody treatment is reported to suppress lung metastasis of cancer cells and to offer a survival advantage to septic mice (7, 8). Downstream intracellular signaling molecules of RAGE include NFκB, ERK (extracellular signal-regulated kinase) 1/2, p38MAPK (mitogen-activated protein kinases), JNK (c-Jun N-terminal kinases), PKC (protein kinase C), Rac/Cdc42, and TIRAP and MyD88, adaptor proteins for TLR 2 and 4 (9). A functional link between RAGE and TLR is thus considered to be in a coordinated manner (10).

Among the above ligands, HMGB1 is known to be readily released from necrotic or damaged cells and to be actively secreted by activated endothelial cells and immune cells such as monocytes, macrophages, dendritic cells, and natural killer cells (11). HMGB1 can form a complex with proinflammatory molecules of CpG DNA, LPS, and interleukin 1β, and this further induces the activation of RAGE signaling (12). HMGB1 is also found to be expressed in human adipose tissues with the expression levels associated with the fat mass and obesity-related genes (13). TLR2 and 4 also recognize HMGB1 and can be involved in HMGB1-induced cellular responses (14). S100 proteins are a family of over 20 proteins that show a structural similarity with their two EF-hand Ca^2+^-binding domains flanked by α-helices. Higher oligomerization states of S100 proteins lead to the activation of RAGE (15). AGE-modified S100A8/A9 have been reported to strongly activate inflammatory responses via RAGE (16). S100A8/A9 was also shown to interact with TLR4 (17). Our groups have also shown that phosphatidylserine on the surface of apoptotic cells and LPS are also RAGE ligands (18, 19). Rapid removal of apoptotic cells by phagocytes is crucial for tissue development, homeostasis, resolution of inflammation, and prevention of autoimmune responses. RAGE was found to function as one of the PS receptors that recognize and initiate apoptotic cell clearance (18). LPS and the lipid A component responsible for LPS toxicity and known as endotoxin were found to directly interact with RAGE (19). LPS is also a well-known TLR ligand.

## RAGE and Adiposity

**Figure 1:**
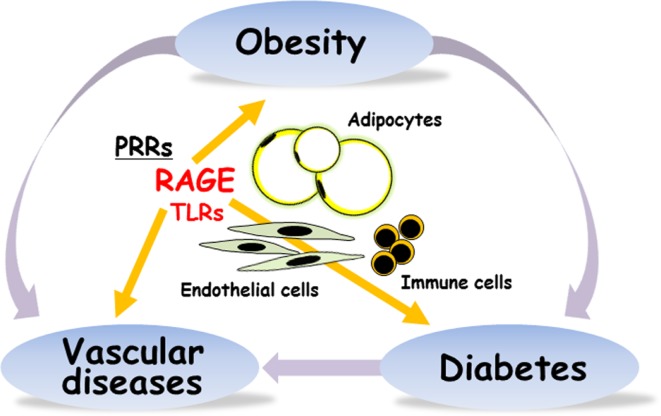
**RAGE is implicated in obesity, type 2 diabetes, and vascular diseases**. PRRs, pattern-recognition receptors; RAGE, receptor for advanced glycation end-products; TLRs, toll-like receptors.

Using RAGE and apoE double deficient mice, Ueno et al. demonstrated that absence of RAGE is associated with decreased epididymal fat weight and smaller adipocyte size, which are significantly associated with the decrease in atherosclerotic lesions (20). They also reported that circulating anti-inflammatory adiponectin levels in apoE^−/−^RAGE^−/−^ were higher than apoE^−/−^RAGE^+/+^ mice, and their levels were significantly and inversely associated with aortic atherosclerosis. Very recently, Monden et al. demonstrated that RAGE directly regulated adipogenesis and hypertrophic process of adipocyte differentiation *in vitro* (21). Adenoviral overexpression of RAGE markedly increased generation of hypertrophic adipocytes and RAGE knockdown by using siRNA system significantly suppressed generation of hypertrophic adipocytes. Under high fat diet feeding in mice, RAGE deficiency is associated with less body weight, less epididymal fat weight, less adipocyte size, higher serum adiponectin, higher expressions of Glut4 and adiponectin in epididymal fat, and greater insulin sensitivity. It is now acceptable that direct role of RAGE in adipocyte hypertrophy and insulin resistance (Figure 1). However, RAGE ligands are still unknown to be involved in the RAGE-dependent adiposity. Further studies are required to characterize the interplays among a variety of RAGE ligands and inflammatory reactions in obesity and type 2 diabetes.

## RAGE Polymorphisms, Obesity, and Inflammation

Several functional single nucleotide polymorphisms have been identified in human RAGE gene. The G82S occurs in the ligand-binding V domain of RAGE and affects ligand affinity, resulting in the enhancement of proinflammatory reactions and immune/inflammatory diseases (22, 23). In obese subjects, S/S carriers showed significantly higher concentrations of AGE and C reactive protein than G allele carrier and lower concentration of soluble RAGE, a decoy receptor (24). S allele at RAGE G82S polymorphism may be more closely associated with proinflammatory reactions under obese conditions rather than non-obese status, thus linking to the development of obesity-associated complications.

We very recently reported that the induction of RAGE expression in pancreatic β-cell by insufficient leptin action under obesity conditions could trigger β-cell failure in type 2 diabetes (25). It is thus considered that RAGE could be a potential targeting receptor for the prevention and treatment of the development of obesity, β-cell failure, vascular complications, and inflammation in type 2 diabetes (Figure 1).

## Conflict of Interest Statement

The authors declare that the research was conducted in the absence of any commercial or financial relationships that could be construed as a potential conflict of interest.
